# Serological responses to a soluble recombinant circumsporozoite protein-VK210 of *Plasmodium vivax* (rPvCSP-VK210) among Iranian malaria patients

**DOI:** 10.1186/s40001-021-00607-6

**Published:** 2021-11-25

**Authors:** Mehdi Nateghpour, Soudabeh Etemadi, Afsaneh Motevalli Haghi, Hamid Eslami, Mehdi Mohebali, Leila Farivar

**Affiliations:** 1 Department of Medical Parasitology and Mycology, School of Public Health, Tehran University of Medical Sciences, Tehran, Iran; 2 Infectious Disease and Tropical Medicine Research Center, Research Institute of Cellular and Molecular Sciences in Infectious Diseases, Zahedan University of Medical Sciences, Zahedan, Iran; 3 Department of Parasitology and Mycology, Faculty of Medicine, Zahedan University of Medical Sciences, Zahedan, Iran; 4 Department of Medical Biotechnology, School of Advanced Technologies in Medicine, Tehran University of Medical Sciences, Tehran, Iran

**Keywords:** *Plasmodium vivax*, Recombinant circumsporozoite protein, VK210, ELISA, Iran

## Abstract

**Background:**

Circumsporozoite protein (CSP) has a central immune domain that includes short regions of repeating amino acid sequences. This immunodynamic region is an epitope of B cells that can elicit an immune response in human and laboratory animals. The aim of the present study was to express the recombinant PvCSP-VK210 antigen and evaluate it for assaying antibodies obtained during human *P. vivax* infection by Western blotting and indirect ELISA (enzyme-linked immunosorbent assay).

**Method:**

Genomic DNA of *P. vivax* was isolated from a blood sample of an Iranian person with vivax malaria, and by PCR, the fragment of the PvCSP-VK210 gene was amplified. The gene fragment was cut after gel purification by BamHI and HindIII enzymes and then cloned into pET28a expression vector. Finally, the recombinant pET28a was transformed into the *E.* *coli* BL21 (DE3) as the expression host. In order to produce His-tagged protein, the expression host was cultured in LB medium. The protein was purified by Ni–NTA columns and immobilized metal affinity chromatography, and after confirmation by Western blotting technique, was used as the antigen in the indirect ELISA test.

**Results:**

The recombinant protein was expressed and purified as a 32-kDa protein. The sensitivity and specificity of the indirect ELISA test with the recombinant PvCSP-VK210 antigen were 61.42% and 97.14%, respectively, based on OD  =  0.313. Between the results of the microscopic test and the indirect ELISA test with the recombinant PvCSP-VK210 antigen there was a Kappa coefficient of 0.586. The positive and negative predictive value and validity of the ELISA test with the recombinant PvCSP-VK210 antigen were 95.55%, 71.57%, 79.28%, respectively.

**Conclusion:**

The sensitivity of the indirect ELISA method with the recombinant PvCSP-VK210 antigen was 61.42%, which is the first report from Iran.

## Introduction

*Plasmodium vivax* infection begins with the entrance of sporozoites into the human host by biting malaria-infected female *Anopheles* mosquito. Sporozoites then migrate to the liver, where they invade hepatocytes and proceed through asexual exoerythrocytic stage development. The parasites develop into schizonts, to start the blood stage development, and some of them remain in dormant hypnozoite form. Hypnozoites can be reactivate and proceed to schizogony process several months later, and their role in malaria relapse [1]. *P. vivax* has an incubation period and direct division of merozoites or re-division from hypnozoites. For this reason, diagnosis of vivax malaria is extremely necessary in asymptomatic, latent stage patients or relapsed infections [2]. Some studies indicate that among the human malaria parasite species, *P. vivax* sporozoite expresses more surface proteins, such as circumsporozoite protein (PvCSP) [2, 3].

Some studies have showed that the central region of PvCSP consists of 15–19 repeats (encoding 9 amino acids), and polymorphism that is the basis for their classification with 3 completely different variants as VK210, VK247 and vivaxLike [3]. Circumsporozoite protein (CSP) has a central immune domain that includes short regions of repeating amino acid sequences. In fact, practical role of the repeat sequence is recognition of species-specific host receptors [4, 5]. The repeat region contains B-cell immunodominant epitopes [6] which is a target for protective antibodies that block the sporozoite invasion of host hepatocytes [4]. Specifically, the central repeat region of *Plasmodium falciparum* CSP, that contains associate immunodominant B-cell epitope, describes the target of the primary vaccine trials [6, 7], while the repeat region of *Plasmodium berghei* CSP alone is not sufficient to mediate sporozoite infectivity in either the mosquito or the mammalian host [8].

Repetitive areas of the VK210 genotype include the combination of GDRA[D/A/P] GQPA amino acid motif. The terminal repeat of the VK210 variant was shown to systematically comprise the GDRAAGQPA amino acid motif that is straight away followed by a conserved GNGAGG post-repeat sequence. The VK247 repeat consists of the same organization of ANGA[G(N/D)]/[DD]QPG motif. Moreover, VK247 terminal repeat systematically comprises the ANGAGNQPG motif that is followed by the conserved ANGAGGQ post-repeat sequence [9, 10]. Antibodies against CSP have been reported to be produced in the early stages of malaria in an infected person and it has been found that the repeat region is contain B-cell immunodominant epitopes, so this protein can be selected as a serological marker for seroepidemiological studies [11].

Although the main, diagnostic tools for detecting vivax malaria parasites, such as microscopic examinations, nested PCR, and rapid diagnostic tests (RDTs) are focused on the blood stage [2], preparing some methods to examine tissue stages can facilitate detecting *P. vivax* in prepatent level [10, 11]. If the anti-CSP antibody response for *P. vivax* (strain Iran) is appropriate and detectable, it can be screened upon the arrival of immigrants in Iran. And if the person has no previous history of the disease and anti-CSP antibody response for *P. vivax* is positive, we can wait for the disease to occur, especially in people who enter Iran from highly contaminated border areas. For analyzing the seroprevalence of CSP antibodies in malaria patients, we tried to clone the VK210 sequence, as dominant repeat sequence in Iranian *P. vivax* strain from the genomic DNA of *P. vivax* patient’s blood and characterized its antigenicity by using Western blot and indirect ELISA, methods in this study.

## Materials and methods

### Study area

Sistan and Baluchestan Province is located in the southeast of Iran. It is between 58 degrees 55 min 63 degrees 20 min east longitude and 25 degrees 4 min 31 degrees 29 min north latitude, and due to its proximity to Afghanistan and Pakistan and high traffic on both sides of the border, marginalization, stagnation of water in some areas, precipitation during Anopheles mosquito activity and high temperature, has an annual incidence of malaria, and given its endemic nature, vivax malaria was selected for this study.

### Blood sample collection

In this study, in order to prepare a recombinant antigen for the indirect ELISA test, patients’ blood sample with *P. vivax* infection, previously confirmed by microscopic examination (thin and thick spread). First step, blood samples with EDTA collected from *P. vivax* infected patients were examined for presence of PvCSP in the sequenced region. Second step, one patients’ blood sample with genotype PvCSP-VK210 was selected for recombinant antigen preparation. 140 blood serum samples were collected for serologic analysis to detect antibodies to the rPvCSP-VK210 antigen. A total of 70 blood serum samples were obtained from patients who were resident in malaria endemic regions in Iran with symptomatic *P. vivax* malaria confirmed by detecting the parasites in thick and thin blood films using Giemsa stain. Negative controls included 70 samples from healthy people without the history of symptomatic malaria infection. However, 12 of these samples were with other infectious disease from patients infected with toxoplasmosis*, Leishmania infantum* infection*,* echinococcosis and falciparum malaria. Blood and serum samples were collected after informed consent was obtained during the period from 2017 to September 2019. The sera were frozen at − 80 °C.

### Preparation of rPvCSP-VK210 antigen

#### Parasite DNA extraction

DNA of the one patients’ blood sample with *P. vivax* infection (genotype PvCSP-VK210) was extracted using High Pure PCR Template Preparation Kit (Roche diagnostics, Germany) according to the manufacturer’s instructions and then kept at − 20 °C until molecular testing processes.

#### Cloning and expression of PvCSP-VK210 in *Escherichia coli*

The PvCSP gene (VK210 variant) was amplified by polymerase chain reaction (PCR) using primers those were designed on the basis of the sequence of *P. vivax* Belem CSP-gene (GenBank: Accession No.: M11926).

F-CSP5′GGA TCC ATG AAG AAC TTC ATT CTC TTG-3′.

R-CSP5-AAG CTT ATT GAA TAA TGC TAG GAC TAA C-3′.

Amplification processes were as follows: initial denaturing step at 95 °C for 5 min; 30 cycles at 95 °C for 30 s, 47 °C for 15 s, and 72 °C for 90 s and final elongation step was conducted at 72 °C for 5 min. These primers contained restriction enzyme sites (BamHI and HindIII enzymes (Thermo scientific) on their 5′ends,) for cloning and expression in the prokaryotic host. pET28a (Novagen, 5369 bp) was digested with sticky-end restriction enzyme. Digestion of plasmids was performed in a 0.5 ml microcentrifuge tube containing; 8 μl plasmid DNA (150ngr), 4 μl of 10X BamHI buffer, 2 μl (10 unit) of BamHI, 2 μl of HindIII and ddH_2_O added up to final volume of 40 μl. A separate set contained 7 μl PCR product (57ngr), 2 μl of 10X BamHI buffer, 1 μl (10 unit) of BamHI and 1 μl of HindIII and ddH_2_O was added up to final volume of 30 μl. The two were mixed by a gentle vortex, centrifuged and incubated at 37 °C for 30 min. Then it was incubated to 80 °C by water bath for 10 min (to inactive the enzyme) to end the digestion**.** The PCR product and the digested pET28a were purified from associate agarose gel using NucleoSpin^®^ Gel and PCR clean-up purification kit (Germany).

The free fragment DNA CSP was ligated into pET28a expression vector that was digested by HindIII and BamHI enzyme. The product of ligation was transformed into *E.* *coli* strain BL21 (DE3), by heat shock. The transformed bacteria were then cultured on Luria–Bertani (LB) agar medium (SIGMA, USA) containing 50 µg/ml kanamycin. The recombinant plasmid was confirmed by each colony PCR and enzyme digestion [12].

PCR screening was carried out by same primers that were used for PCR amplification. A single positive clone was selected for CSP expression, and injected into 20 ml culture tube containing LB broth medium and 50 µg/ml kanamycin and allowed to grow at 37 °C on a shaker at 180 rpm, overnight. The next day, it was sub-cultured into two 100-ml flasks LB broth medium (SIGMA, USA) with 50 µg/ml kanamycin and then, incubated at 37 °C with shaking at 200 rpm for 3 h to reach OD 0.4, one mM of isopropyl β-d-1-thiogalactopyranoside (IPTG) as a gene expression inducer was added to the culture and the culture was continued for overnight. After incubation, the culture was centrifuged at 4000 rpm for 20 min at 4 °C and added to lysis buffer precipitate [0.121 gr Tris–HCl, 4 M urea, 0.0015 g dithiothreitol (DTT), and 200 μl of Triton 100  ×  and ddH_2_O up to 10 ml] and was lysed during five cycles of freeze-thawing. The bacterial lysate was centrifuged at 13,000 rpm for 20 min at 4 °C and the supernatant was used to confirm the expression by SDS-PAGE. Gel was stained with Coomassie blue.

#### Protein purification and Western blotting technique

Using the Bug Buster Ni–NTA His•Bind Purification Kit (Novagen), the recombinant His-tagged protein was isolated from the supernatant of bacterial lysate through the nickel-chelating resin column. During each purification step, proteins were analyzed with the sodium dodecyl sulfate-polyacrylamide gel electrophoresis (SDS-PAGE). The Bradford test used spectrophotometer (Meylan, France) to calculate the protein concentration.

The separated proteins were transferred to 0.45 μm PVDF membranes (Millipore, Billerica, MA, USA) in a semi-dry transfer buffer (25 mM Tris, 192 mM glycine, 20% methanol) at constant voltage (70 v) for 90 min using a semi-dry blotting system (Bio-Rad Germany). After blocking with 2.5% skim milk and glycerol 2.5% in Tris’ buffer saline containing 0.05% Tween 20 (TBS/T). Followed by anti-poly histidine HRP from mouse (Sigma) diluted in TBS/T (1:2000), 10 vivax malaria poled serum and sera from healthy individuals diluted to TBS/T (1:100) were used for the capture. A secondary anti-human IgG–peroxidase (horseradish peroxidase (HRP) conjugated rabbit anti-human IgG) (1:1000, TBS/T) (Sigma-Aldrich USA) was used to detect His-tagged recombinant proteins. The strips were visualized at room temperature for 15 min following color production in DAB substrate solution. The reaction was halted by washing into diluted H2O four times.

#### Indirect ELISA

Indirect ELISA tests were performed to confirm whether antibodies to the rPvCSP-VK210 antigen were present in the blood samples. Briefly, rPvCSP was tittered using a checkerboard titration with 100 μl of rPvCSP serially diluted flat-bottom plates varying from the initial 11 μg /ml protein concentration in the sodium carbonate buffer pH 9.6 (coating buffer) and incubated in a wet chamber at 4 °C overnight. The wells were washed three times with phosphate buffer saline containing 0.05% Tween 20 (PBS-Tween 20).

To each previously coated well, known positive and negative sera diluted to 1:20, 1:40, 1:80, and 1:160 in PBS-T were added serially. The plates were incubated in a wet chamber at room temperature for 90 min along with shaking, then washed with PBS-T 5 times. Peroxidase-conjugated anti-Human IgG (Sigma, 1:1,000 vol/vol) diluted in PBS-T buffer was then added. The plates were re-incubated in a wet chamber for one hour at room temperature along with shaking then washed with PBS-T 5 times.

Following this step, color production was achieved by adding 100 μl O-phenylenediamine (dihydrochloride) substrate (SIGMA) to the plates and then incubated in a dark place together with shaking for 15 min. The OPD reaction was stopped with 50 µl (3 N HCl or 3 M H2SO4) solution, and read at 490 nm using ELISA plate reader (MTX Lab Systems Canada). The cut-off value for positivity was set at the mean  +  2 standard deviations from the negative control samples. All assays were double-checked. Eventually, the most successful antigen and antibody concentrations were determined, and the planned indirect ELISA was applied in natural infections to test gift antibodies.

#### Statistical analysis

The optical density (OD) values were considered positive more than the mean  +  2SD values.

In this study, the microscopy method for the peripheral blood was considered as the golden standard. The sensitivity, specificity, and compatibility of the indirect ELISA test with the recombinant rPvCSP-VK210 antigen were compared by those of the microscopic test. Sensitivity, specificity, positive and negative predictive values, and validity were used to compare the tests. Kappa coefficient was used to determine the agreement between indirect ELISA test with the recombinant antigen and the microscopy method. SPSS software version 20 was used to calculate the kappa coefficient.

## Results

### Cloning and expression of rPvCSP-VK210

**Fig. 1:**
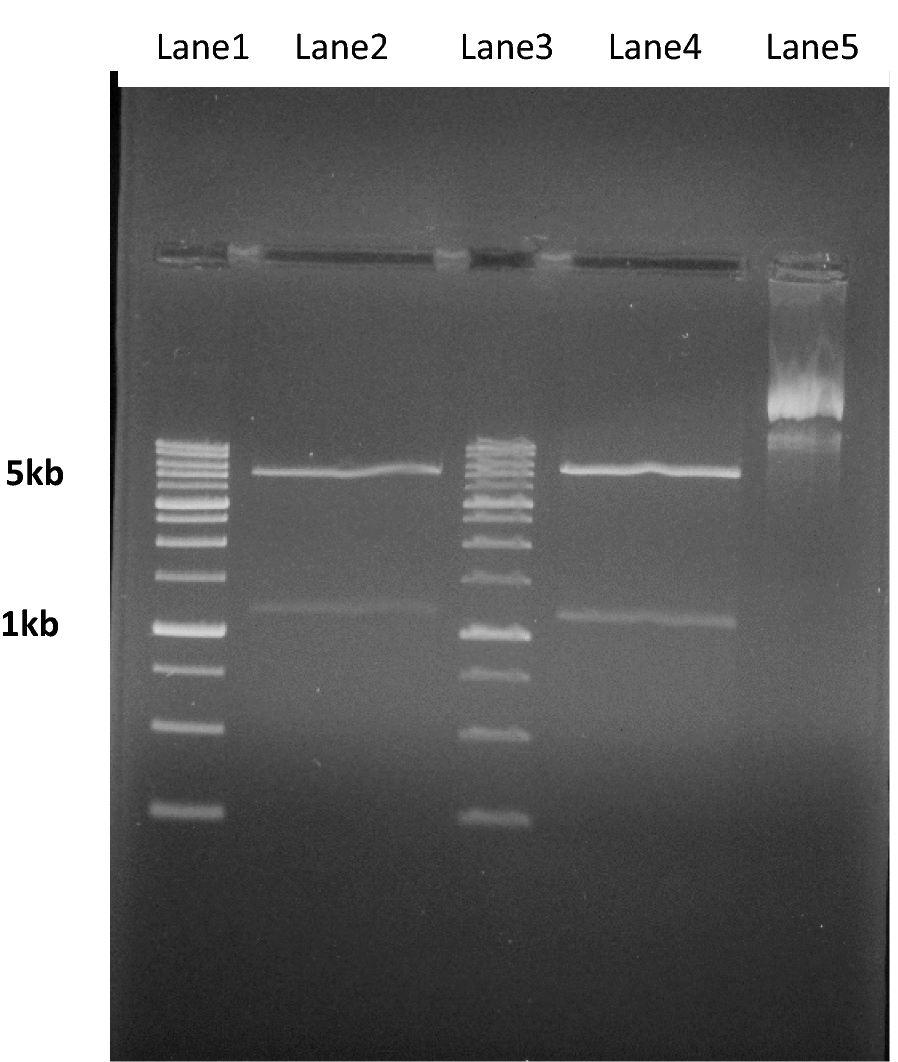
Undigested pET28a/CSP-VK210, digested recombinant pET28a/CSP-VK210 by BamHI and Hinlll restriction enzymes and expected insert band are shown. Lanes 2, 4: separated insert from digested recombinant expression plasmid (pET28a/CSP-VK210) 1037 bp and digested recombinant expression plasmid 5300 bp approximately. Lane 5: intact recombinant expression plasmid, lanes1, 3: size marker: 1 kb

**Fig. 2:**
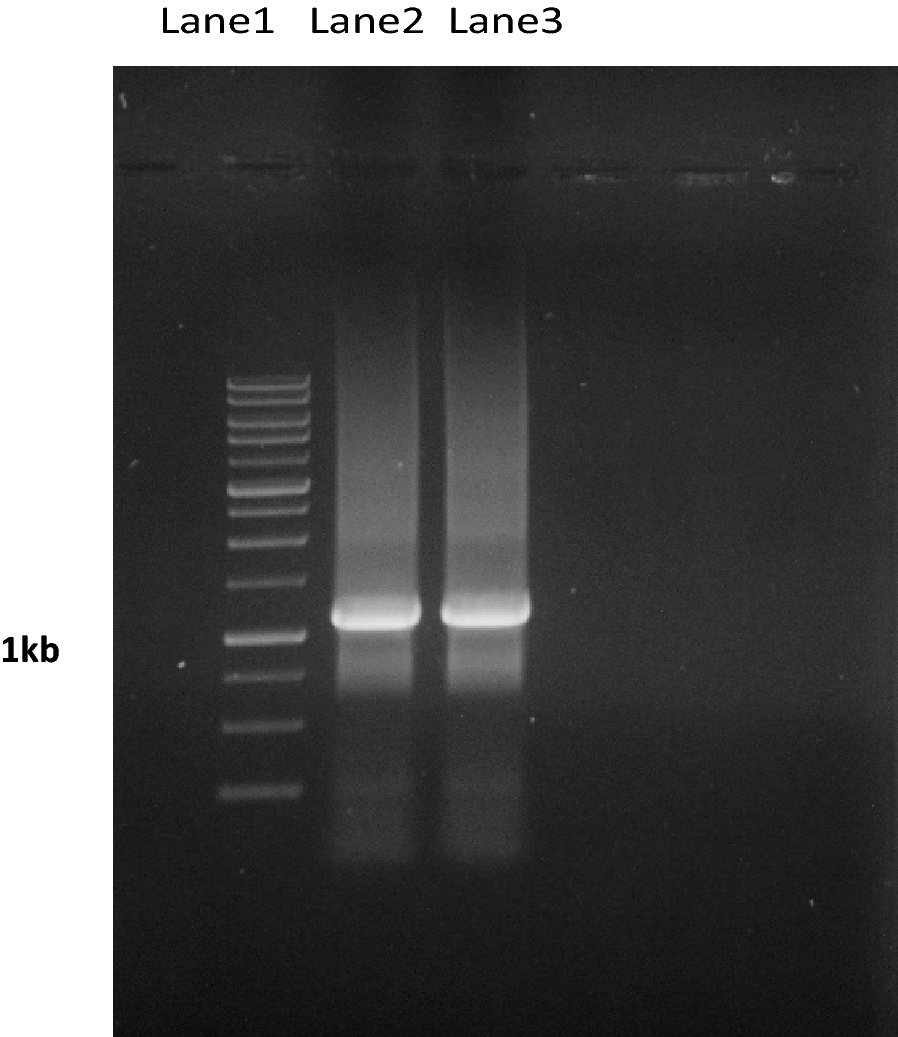
Colony and PCR results using primers designed on recombinant plasmids and duplication of the fragment 1037 bp. Lane 1: size marker 1 kb. Lanes 2, 3: amplification fragment inserts 1037 bp

The intron-free PvCSP-VK210 gene fragment was approximately 1100 bp after amplification (Fig. 1). The results of enzymatic cutting and sequencing and colony PCR all indicated cloning success. (At 37 °C, the induction with 1 mM IPTG and precipitation for 6 h after induction) (Fig. 2).

**Fig. 3:**
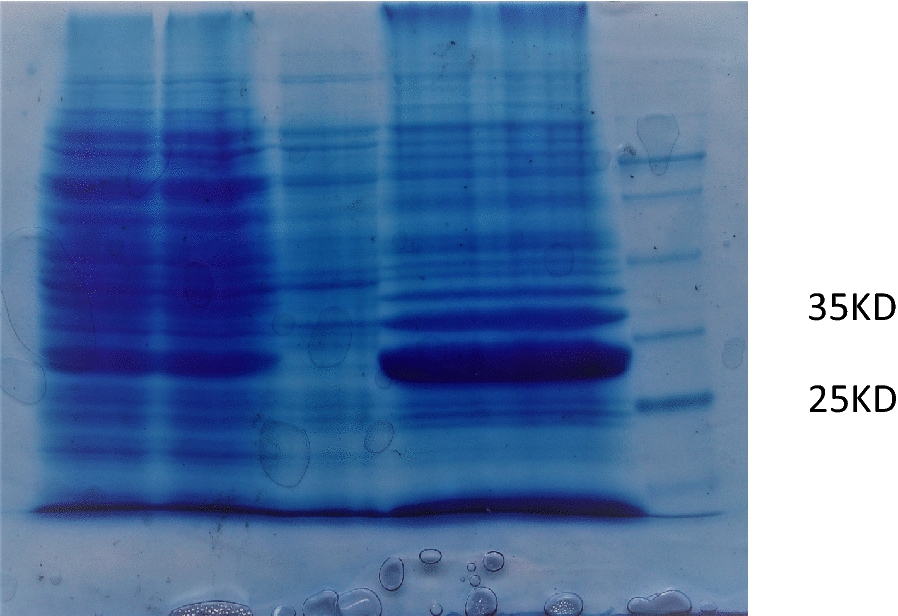
SDS-PAGE analysis on the expression of pET28a/PvCSP-VK210 in *E.* *coli* (BL21). Lanes 1, 2, *E.* *coli* BL21(DE 3) supernatant containing pET28a/PvCSP-VK210 recombinant plasmid (lysis buffer contains 4 M urea). Lane 3, *E.* *coli* BL21(DE 3) bacterial lysate containing pET28a plasmid (negative control). Lanes 4, 5 *E.* *coli* BL21(DE 3) sediment containing pET28a/PvCSP-VK210 recombinant plasmid (lysis buffer contains 4 M urea). Lane 6, protein size marker

After cloning and sequencing and confirmation of the existence of the target fragment in the vector, the presence of a stop codon at position 912 caused the fragment length to be changed to 885 nucleotides. And the protein fragment contained 285 amino acids with an approximate weight of 32 kDa (Fig. 3).

This protein consisted of two domains, the domain I having a five amino acid sequence (KLKQP), and the central repeat domain comprising 14 sequences of 9 amino acids (rapid tandem). These duplicates included G D R A D G Q P A and G D R A A G Q P A. The alteration in the present protein had no effect on the working process. Since the original target was to clone the central repeat region (PvCSP-VK210 genotype specific region) from the beginning, and because the central repeat domain and domain I were present in the protein, work continued.

### Reactivity of *P. vivax* infected serum samples to rPvCSP-VK210

**Fig. 4:**
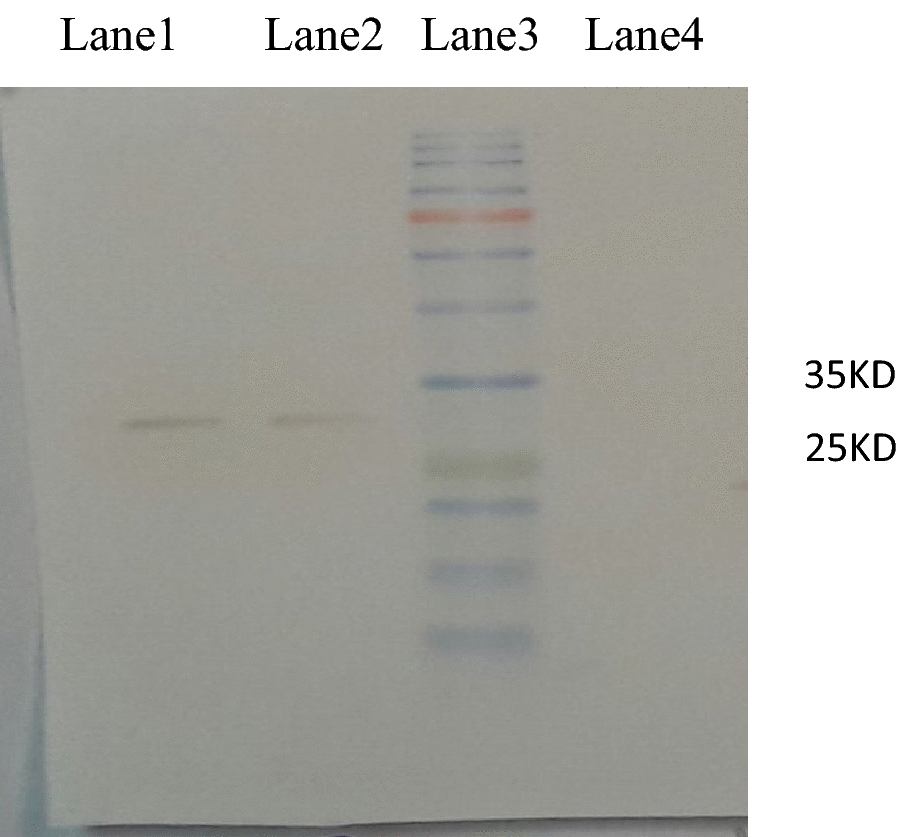
Western blot analysis of His-tag/PvCSP-VK210. Samples were run on 12% polyacrylamide-SDS gels and then transferred to nitrocellulose. Lane 1, His-tag/PvCSP-VK210 reacted with an anti-His antibody, lane 2, His-tag/PvCSP-VK210 reacted with 5 human *P.* *vivax* positive serum (1:100 dilution); lane 3, protein size marker; lane 4, purified PvCSP-VK210 that did not react with human *P.* *vivax* negative serum

Immunoblotting test results showed that the 32 kDa brown bands, which were the result of the interaction of the recombinant protein from overnight culture after induction with 1 M IPTG, reacted with 10 different serums from those infected with *P. vivax*, but with the control serums (healthy subjects who had no symptoms of vivax malaria and their microscopic test was negative) had no reaction. Also, to confirm the expression of the recombinant protein due to the presence of histidine at the N terminus, anti*-*polyhistidine with a dilution of 1:2000 was used, and this dilution of anti*-*polyhistidine reacted with the histidine of the recombinant protein, and a 32-kDa band appeared (Fig. 4).

### Calculation of appropriate concentration of antigen and sera

From the results of the checkerboard test, the most effective concentration for antigen was defined as 0.5 μg/ml of rPvCSP-VK210 of sodium carbonate buffer, pH 9.6 in each well, and also the best dilution to detect malaria antibody was determined as 1/40 dilution. All examined sera were diluted at an optimal dilution of 1/40 and added to the ELISA plates coated with rPvCSP-VK210. Human antibodies against rPvCSP-VK210 were detected by an indirect ELISA. The very best dilution that created an absorbance value over the highest absorbance of the negative control was considered as the best point concentration to assess presence of the antibodies in sera. The cut-off point for rPvCSP-VK210 was calculated (Tables 1, 2).

**Table 1:** Calculation of suitable concentration of antigen and sera

Positive sera				Negative sera				
Sera dilution	1/20	1/40	1/80	1/160	1/20	1/40	1/80	1/160
Dilution Ag (μg/ml)	1	2	3	4	5	6	7	8
0.5	0.915	0.953	0.633	0.566	0.392	0.201	0.206	0.207
0.25	0.860	0.740	0.627	0.582	0.233	0.252	0.251	0.238
0.125	0.822	0.654	0.519	0.441	0.266	0.298	0.320	0.297
0.062	0.867	0.701	0.745	0.691	0.267	0.263	0.280	0.284

The best titration result for the antigen concentration was 0.5 μg/ml, for the human sera it was a 1:40 dilution and for the IgG conjugate it was a 1:1000. The numbers 1–8 represent the wells of ELISA plates

**Table 2:** Determination of IgG cut-off from traditional community survey

	A	B	C	D	E	F	G	H
1	0.177	0.220	0.187	0.170	0.193	0.188	0.215	0.177
2	0.137	0.163	0.148	0.160	0.222	0.176	0.135	0.265
3	0.144	0.161	0.173	0.244	0.199	0.120	0.199	0.237
4	0.242	0.082	0.289	0.296	0.233	0.089	0.288	0.111
5	0.151	0.180	0.113	0.137	0.194	0.279	0.136	0.144
6	0.303	0.140	0.153	0.129	0.154	0.220	0.187	0.223
7	0.105	0.079	0.105	0.205	0.390	0.356	0.213	0.219
8	0.137	0.228	0.150	0.188	0.244	0.242	0.216	0.161
9	0.232	0.184	0.213	0.164	0.146	0.044		

MN + 2SD	(Standard deviations) SD	MN	Number of samples
0.313	0.063	0.187	70

The cut-off point for with rPvCSP-VK210 was calculated as (OD490  = 0.313 for IgG). The rows of 1–9 and columns of A–H represent the wells of ELISA plates

### Determination of clinical sensitivity and specificity of indirect ELISA

As shown in Table 3, the results indicated that 38.57% (27/70) of sera from confirmed *P. vivax* infected subjects did not react with rPvCSP-VK210 and 2.85% (2/70) of healthy subjects reacted with rPvCSP-VK210. Although the rPvCSP-VK210 showed reactivity with two samples from healthy subjects, cross reaction was also observed with *P. falciparum*. Indirect ELISA results showed clinical sensitivity of 61.42% for patients with confirmed malaria vivax infection and the specificity was determined as 97.14%. The positive predictive value (PPV), the negative predictive value (NPV) and the diagnostic efficiency of anti-rPvCSP-VK210 antibody using indirect ELISA were determined as 95.55, 71.57 and 79.28%, respectively (Table 4). Comparison between seropositive and seronegative individuals with *P. vivax* infection using indirect ELISA test in the case and the control groups showed a statistically significant difference (*P*  < 0.05).

**Table 3:** Diagnostic performance of anti rPvCSP-VK210 indirect ELISA assay and comparison between ELISA using recombinant protein rPvCSP-VK210 (cut-off  = 0.313) and microscopy results

rPvCSP-VK210 indirect ELISA	Positive ELISA result		Negative ELISA result		Total	
Microscopic examination	Number	Percentage	Number	Percentage	Number	Percentage
Positive microscopic result	43	61.42	27	38.57	70	100
Negative microscopic result	2	2.85	68	97.14	70	100
Total	45	32.14	95	67.85	140	100

**Table 4:** **Diagnostic** performance of anti-PvAMA-1 indirect ELISA assay

Assay	Sensitivity %	Specificity %	PPV %	NPV %	Diagnostic efficiency
ELISA	61.42	97.14	95.55	71.57	79.28

## Discussion

Malaria is one of the most common parasitic diseases in the tropical regions of the world, with more than 214 million new cases per year [13]. In spite of the high burden of the disease, infections of *P. vivax* were commonly ignored because it produces a relatively benign malaria, but now it has been determined that *P. vivax* can cause severe disease similar to *P*. *falciparum* [14]. With this in mind, attempts to handle these problems through development of vaccine, newer diagnostics and accelerated care tools aiming at global eradication of malaria are increasing [15]. Currently, two vaccine candidates for *P. vivax*; Duffy-binding protein (DBP) and CSP gene have been tested in clinical trials compared to 23 vaccine candidates for *P*. *falciparum* [16]. Several factors such as difficulties in culture, limited animal models for *P. vivax*, other technical challenges including protein function, antigenic diversity, and inhibition of growth in laboratory conditions prevent the selection of suitable antigens for *P. vivax* studies [17]. Researchers have recognized that the central repeat regions of the CSP in *P. vivax* are epitopes of B cells, and specific antibodies to this region can protect a person against malaria by blocking the attack of sporozoites to the liver cells [18].

Therefore, we proposed to design a serology marker for *P. vivax* that was the main purpose of this study and considering that genotype vk210 is the dominant type in Iran, it was used for preparing the recombinant antigens and serological studies in our study. Sporozoite antigens, invasive merozoite antigens, infectious red blood cell (RBC) antigens and gametocyte antigens were the potential targets of antibodies in various studies and have been investigated as serologic markers in response to IgG and IgM antibody [19]. In two cross-sectional and case–control studies conducted in Turkey and Brazil, respectively, with the CSP serology marker, the sensitivity of IgG antibody response to VK210 and VK247 alleles reported in Brazil were 74% and 57%, respectively, while the cross-sectional study in Turkey reported a 2.4-fold increase in *P. vivax*’s infection in response to IgG antibody to the combination of two alleles, VK210 and VK247 [20, 21]. In the present study, ELISA test showed 61.42% clinical sensitivity in patients with confirmed malaria vivax infection and the specificity was 97.14%. This indicates that the IgG antibody response is greater than the combination of two alleles and can detect more cases of infection. A study in South Korea reported that the recombinant antigens as separate alleles for both VK210 and VK247 have sensitivity of 47.9% and 57% for IgG antibodies, respectively [22]. In the present study, 27 false negative and 2 false positives were detected in vivax infected patients and healthy samples, respectively. Although the explanation of this phenomenon was still unclear, it is not infrequent for patients exposed to *P. vivax* infection not show anti-CSP antibodies, particularly if the infection was caused by relapse episode. Recently, lower immunogenicity of VK247 peptide had been analyzed [22] and antibodies to VK210 were more frequent than those to VK247 [3]. Because of the differences in immunogenicity, the prevalence of VK247 was not as high as VK210 type in endemic countries [23]. Study of Pyo-yuncho et al. in South Korea using a CSP recombinant antigen and ELISA test showed that there was a strong correlation between the annual parasite incidence (API) and the rate of antibody response to the CSP antigen, which indicated that the immune response increased according to age [24]. Meanwhile, report by Yang et al. indicated that there was a negative correlation tendency between the number of repeat and immune responsiveness [25], So, it can be said that increasing the number of repeat sequences does not necessarily have significant effect in the preparation of recombinant antigens.

Protein structure and amino acid sequence of the recombinant rPvCSP-VK210 molecule showed that region I, includes amino acids (KLKQP) and central repeat region (GQPAGDRAA, GQPAGDRAD) which are B and T cells epitopes. The present study, in trying to identify the human naturally acquired immune responses to rPvCSP-vk210 antigen representing the 2 domains of PvCSP gene in Iran, a recombinant plasmid, pET28a-CSP, was created and also the connected protein was used as a coated antigen in ELISA plates. Our study was able to provide a suitable expression vector with a possible power to express the CSP gene in prokaryotic cells, and so, it will be simply available to organize related recombinant protein in great amount within the future. Another benefit of this study was the use of an expression vector without the need for a cloning vector, clear evidence for cost reduction and time saving. The sensitivity of ELISA evaluated with sera from naturally infected people was 61.42% and the specificity value of the ELISA determined with sera from healthy subjects and from people with different infectious diseases was 97.14%. The positive predictive value (PPV), the negative predictive value (NPV) and also the diagnostic efficiency of anti-rPvCSP-VK210 antibody using indirect ELISA were 95.55, 71.57 and 79.28%, respectively. Similar studies in Iran (preparation of recombinant antigen of *P. vivax*) including Mirahmadi et al. (who used recombinant merozoite surface antigen-142 kDa of *P. vivax*) and Motevalli et al. [who used recombinant *P. vivax* apical membrane antigen-1 (rPvAMA-1)] reported immune responses to recombinant antigen of *P. vivax*, different sensitivities of 81% and 86.9%, respectively [26, 27].

Increased IgG antibody response against recombinant DBP2 antigen of *P. vivax* (Souza-Silva et al. [28]) and recombinant MSP antigen of *P. vivax* (Lima-Junior et al. [29]) have been reported and the results show that these antigens are exposed to the study population and increase the chance of *P. vivax* infection [28, 29].

Although microscopic testing of malaria detection is a gold standard for diagnosis of malaria, its disadvantages include the need for skilled and trained persons, time consuming and loss of patients with low parasitism. Therefore, detection of antibodies, which requires use of diagnostic kit for quick diagnosis of the antibodies can compensate for the disadvantages of microscopic testing. While most malaria patients have malaria antibodies, even with a low level of parasitemia, hence serologic methods based on antigen/antibody detection can be used for extensive epidemiological studies. During the biological process of malarial infection, exposure of CSP to antigen–antibody reaction up to 61.42.0% is that the first report in Iran.

## Data Availability

That if the paper contains material (data or information in any other form) that is the intellectual property and copyright of any person(s) other than the author(s), then permission of the copyright owner(s) to publish that material has been obtained, and is clearly identified and acknowledged in the text of the paper.
